# Unraveling the Role of Cuticular Protein 3-like (HvCP3L) in the Chitin Pathway through RNAi and Methoxyfenozide Stress Response in *Heortia vitessoides* Moore

**DOI:** 10.3390/insects15050362

**Published:** 2024-05-16

**Authors:** Hanyang Wang, Mingxu Sun, Na Liu, Mingliang Yin, Tong Lin

**Affiliations:** College of Forestry and Landscape Architecture, South China Agricultural University, 483 Wushan Road, Guangzhou 510642, China; frisk990709@gmail.com (H.W.); isunmingxv@icloud.com (M.S.); ln021994@163.com (N.L.); mingliang.yin@scau.edu.cn (M.Y.)

**Keywords:** *Heortia vitessoides* Moore, cuticular proteins, RNA interference, chitin pathway, methoxyfenozide stress, growth and development

## Abstract

**Simple Summary:**

The insect epidermis covers multiple parts of the insect body, fulfilling both structural and protective roles. Primarily composed of chitin and epidermal proteins, the insect epidermis displays considerable diversity among insects. The CPR family, the largest and most diverse group of cuticular protein (CP) genes identified to date, plays crucial roles in insect development, including body length regulation and cuticle formation. Steroid hormones, such as ecdysteroids, regulate the expression of epidermal proteins. In addition, the insecticide Methoxyfenozide mimics the action of 20E, thereby affecting insect growth. Our study on *Heortia vitessoides* Moore elucidates the role of HvCP3L and offers novel insights into pest management strategies for *Aquilaria sinensis* (Lour.) Spreng.

**Abstract:**

Cuticle proteins (CPs) constitute a multifunctional family; however, the physiological role of Cuticle Protein 3-like (CP3L) in *Heortia vitessoides* Moore remains largely unclear. In this study, we cloned the HvCP3L gene from the transcriptional library of *Heortia vitessoides* Moore. RT-qPCR results revealed that HvCP3L exhibited high expression levels during the larval stage of *Heortia vitessoides* Moore, particularly at the L5D1 stage, observed in both larval and adult heads. Through RNA interference, we successfully silenced the HvCP3L gene, resulting in a significant reduction in the survival rate of *Heortia vitessoides* Moore, with the survival rate from larvae to adults plummeting to a mere 17.7%, accompanied by phenotypic abnormalities. Additionally, we observed that the knockdown of HvCP3L led to the inhibition of genes in the chitin pathway. Following exposure to methoxyfenozide stress, the HvCP3L gene exhibited significant overexpression, coinciding with phenotypic abnormalities. These findings underscore the pivotal role of HvCP3L in the growth and development of *Heortia vitessoides* Moore.

## 1. Introduction

The insect epidermis covers the exterior of the insect body, encompassing the thorax, foregut, midgut, hindgut, and the inner lining of the trachea. It not only determines the insect’s physical morphology but also serves as a site for muscle attachment, forming a protective layer against water loss and pathogen intrusion. Furthermore, pigment deposition within the epidermis results in a diverse array of colors and patterns, aiding in camouflage and reducing the risk of predation [1,2]. The primary constituents of the insect epidermis are chitin and epidermal proteins, which interact to create the Bouligand model, enhancing the stability of the epidermis’s intricate structure and maintaining its elasticity and other physical properties [3].

In 1982, the sequences of four cuticular proteins were obtained from the *Drosophila melanogaster*, marking the first discovery of gene sequences encoding cuticular proteins [4]. With the advancement of insect genome sequencing, the genomes of insects such as *Drosophila melanogaster*, *Anopheles gambiae*, *Bombyx mori*, *Apis mellifera*, and *Tribolium castaneum* have been sequenced successively, leading to the identification of a large number of cuticular protein genes.

Insects represent a highly diverse group, exhibiting variations in the number and characteristics of their cuticular proteins, which significantly influence the structure and properties of the insect cuticle. On average, cuticular proteins account for approximately 1% of the total protein-coding genes in insects, demonstrating different types and expression levels across various developmental stages and tissues [5,6,7].

Annotation of cuticle protein genes reveals significant differences in both the types and quantities of CPs genes among different species. Among these, the CPR family stands out as the largest and most diverse group of CPs genes identified to date, characterized by the presence of the Rebers and Riddiford consensus structure (R&R Consensus). Based on variations in the type and location of the cuticular layer, the CPR family can be subdivided into three subfamilies: RR-1 (predominantly found in flexible cuticles), RR-2 (predominantly found in rigid cuticles), and RR-3 [8,9]. The CPAP family encompasses the chitin-binding domain ChtBD2 and can be further divided into CPAP1 (containing one ChtBD2 binding domain) and CPAP3 (containing three ChtBD2 binding domains) [10]. The CPLC family belongs to the low-complexity protein family, with members including CPLCG containing glycine, CPLCW containing tryptophan, CPLCP containing proline, and CPLCA containing alanine, with CPLCW and CPLCG predominantly found in mosquitoes [11,12,13]. The CPCFC family’s conserved domain contains the C-X5-C motif, repeated 2–3 times [14]. The CPF family contains domains composed of 42–44 conserved amino acids, while the CPFL family shares homology with the CPF family but lacks domains, having only a conserved C-terminal [15]. The Tweedle family was discovered in *Drosophila melanogaster* in 2006, named after the mutant phenotype of one of its members [16]. In 2015, a new cuticular protein family named the 18aa family was discovered in *Manduca sexta* [17]. Similarly, in 2016, a new cuticular protein family was also found in *Bombyx mori* [18]. However, some cuticular proteins cannot currently be classified into known families. With the development of bioinformatics, it is anticipated that more new cuticular protein families will be discovered in the future.

To date, a significant body of research on the functional roles of insect cuticular protein genes has demonstrated their crucial involvement in cuticle formation. For instance, in studies on *Drosophila melanogaster* body morphology, the absence of the cuticular protein gene TweedleD1 was found to result in visibly shortened body length in both larvae and pupae, indicating the participation of such cuticular protein genes in shaping insect body form [16]. Additionally, the loss of the RR-1 type cuticular protein gene BmorCPR2 in *Bombyx mori* led to a significant increase in intersegmental folds in larval body segments, restricting cuticle extension and causing an increase in internal cavity pressure, resulting in conspicuous protrusions at the end of each larval segment and between segments, ultimately leading to deformities [19]. Silencing of cuticular protein genes TCPAPI-C, TeCPAP1-H, and TCPAP1-J in *Tribolium castaneum* revealed that, although larvae successfully pupated, slow development from the pupal to adult stage, coupled with incomplete cuticle and elytron development, ultimately led to complete mortality [10]. In studies on *Tribolium castaneum*, silencing of genes TcCPR27 and TcCPR18 resulted in shortened elytra in adults, accompanied by wrinkling, bending, and porous structures, and death approximately one week after eclosion [20]. In *Bactrocera dorsalis*, injection of dsRNA targeting four cap3 genes significantly delayed larval-to-pupal development, with pupation time significantly longer than the control injection [21]. In *Locusta migratoria*, the absence of LmCP8 affected the structural development of the ovipositor, resulting in a large number of unproduced egg capsules being retained in the locust’s ovaries [22]. These studies collectively demonstrate the crucial role of cuticular protein genes in the growth and development of insects. However, the function of cuticular proteins in *Heortia vitessoides* Moore remains unclear.

Cuticular protein genes exhibit diverse expression patterns in various tissues and at different developmental stages, primarily regulated closely by hormones such as ecdysteroids, such as 20-hydroxyecdysone (20E). During insect metamorphosis, the structure and composition of the cuticle undergo drastic changes, and cuticular protein genes, as essential components of the cuticle, are regulated by insect hormones. Studies on the expression patterns of cuticular protein genes in *Bombyx mori* have revealed that most cuticular protein genes are highly expressed when ecdysteroid titers decrease or are undetectable, indicating a negative correlation between the expression of cuticular protein genes and ecdysteroid titers [23]. In the ecdysone signaling pathway, E75 contributes to the promotion of 20E biosynthesis. Knockdown of E75 in *Tribolium castaneum* results in the suppression of TcCPR27, indicating that this cuticular protein gene is positively regulated by the 20E signal [24]. However, there are also some cuticular protein genes whose expression is positively correlated with ecdysteroid titers, such as cuticular proteins TmACP20 and TmACP22 in *Tenebrio molitor* studies [25,26].

Methoxyfenozide belongs to the hydrazide class of insecticides, acting as an antagonist of 20-hydroxyecdysone (20E). It accelerates the molting process in larvae, leading to premature molting, and exhibits high insecticidal activity against Lepidopteran pests [27,28]. Despite its relatively short persistence, methoxyfenozide still demonstrates significant efficacy in pest management. Compared to traditional chemical pesticides, methoxyfenozide has lower ecological toxicity and higher safety for non-target organisms [29,30,31]. In this experiment, we used methoxyfenozide to simulate the exogenous action of 20E, aiming to investigate its effects on the growth and development of *Heortia vitessoides* Moore. This study provides deeper insights into the impact of methoxyfenozide on insects, offering scientific grounds for further pest management strategies.

*Aquilaria sinensis* (Lour.) Spreng. (Myrtales: Thymelaeaceae: Aquilaria) is an important economically valuable evergreen tree [32,33]. However, *Heortia vitessoides* Moore, as a severe foliage-feeding pest, has caused long-term damage to *Aquilaria sinensis* (Lour.) Spreng in the Chinese agarwood zone, characterized by extensive leaf consumption within a short period. Interestingly, the sole food source of *Heortia vitessoides* Moore is *Aquilaria sinensis* (Lour.) Spreng [34]. This study employed the RT-qPCR method to assess the specificity of HvCP3L gene expression at different times and in various tissues. Subsequently, through RNAi technology, the role of the HvCP3L gene in the growth and development process of *Heortia vitessoides* Moore was analyzed, along with its impact on the chitin synthesis pathway. Additionally, we determined the expression levels of the HvCP3L gene after methoxyfenozide treatment, further confirming the significance of this gene in insect growth and development. Through this research, we provide a scientific basis for exploring molecular biology methods to control *Heortia vitessoides* Moore.

## 2. Materials and Methods

### 2.1. Insects

*Heortia vitessoides* Moore specimens were collected from Tianluhu Forest Park in Guangzhou (latitude 23°15′ N, longitude 113°25′ E). Upon bringing the insects back to the laboratory, they were reared on agarwood leaves in a controlled environment chamber set at a temperature of (26.0 ± 1.0) °C and a humidity of 70% ± 10%, with a photoperiod of 14 h of light and 10 h of darkness. The rearing protocol followed standard practices. Mature larvae were transferred to sandy soil boxes with a humidity of 50% ± 10% to facilitate pupation and eclosion. Adults were provided with a 7% honey solution for nourishment.

### 2.2. Sample Preparation

To investigate the expression characteristics of the target gene in different developmental stages of *Heortia vitessoides* Moore, we selected 90 L1 larvae (three biological replicates, with 30 larvae each), 45 L2 larvae (three biological replicates, with 15 larvae each), six larvae each of L3, L4, L5D1-L5D4 stages (three biological replicates, with two larvae each), six pupae, and six adults (three biological replicates, with two individuals each) for analysis. Additionally, to examine the tissue-specific expression of the target gene, we dissected L5D1 larvae into head, epidermis, fat body, foregut, and hindgut; 1-day-old male and female adults were dissected into head, thorax, legs, abdomen (classified by sex), and wings. Furthermore, we selected well-developed larvae of uniform body size from the L4 stage for RNAi and subsequent stress experiments. All these samples were gently wiped with sterile cotton balls, rapidly frozen in liquid nitrogen, and stored at −80 °C for subsequent RNA extraction and cDNA synthesis.

### 2.3. Sequence Verification and Phylogenetic Analysis

We searched the *Heortia vitessoides* Moore transcriptome database using the keyword “cuticular protein 3-like” and identified all single gene clusters annotated as cuticular protein 3-like. These sequences were subjected to nucleotide (Nucleotide BLAST) and protein (BLASTx) comparisons on NCBI (https://blast.ncbi.nlm.nih.gov/Blast.cgi (accessed on 21 May 2023)) to select the most complete sequence of the cuticular protein 3-like gene in *Heortia vitessoides* Moore. This sequence was designated as HvCP3L (Accession number: PP468568). The cDNA sequence of the open reading frame (ORF) of HvCP3L was obtained using the ORF finder tool (http://www.ncbi.nlm.nih.gov/gorf/gort.html (accessed on 27 May 2023)). Specific primers for amplifying the HvCP3L gene were designed using Primer Premier 5.0 software (Premier Biosoft International, Palo Alto, CA, USA), and PCR amplification conditions were set as follows: 98 °C for 3 min; 15 cycles of 98 °C for 20 s, 66 °C for 10 s, and 72 °C for 15 s (with a decrease of 1 °C per cycle); followed by 25 cycles of 98 °C for 20 s, 52 °C for 10 s, and 72 °C for 15 s, with a final extension at 72 °C for 2 min, and then held at 12 °C. The amplified product was purified and sequenced to confirm successful cloning of the target gene.

Furthermore, the ExPASyProtParam tool (http://web.expasy.org/protparam/ (accessed on 12 June 2023)) was used to predict the isoelectric point and relative molecular weight of HvCP3L, while the NetNGlyc 1.0 Server (https://services.healthtech.dtu.dk/services/NetNGlyc-1.0 (accessed on 15 June 2023)) was employed to predict potential transmembrane domains in the protein sequence. The Jpred online software (http://www.compbio.dundee.ac.uk/jpred/index.html (accessed on 15 June 2023)) was utilized for secondary structure prediction of the cuticular protein HvCP3L. Amino acid sequences of cuticular protein 3-like from other insects were retrieved from NCBI, and a phylogenetic tree was constructed using the neighbor-joining method implemented in MEGA 7.0 software (MEGA Limited, Auckland, New Zealand).

### 2.4. RNA Extraction and cDNA Synthesis

Total RNA was extracted from the samples using the Total RNA Kit II (OMEGA). Subsequently, the concentration of the extracted RNA was measured using an Implen NanoPhotometer (NanoPhotometer series (Implen, Munich, Germany)). cDNA synthesis was performed using the PrimeScript^TM^ RT Reagent Kit (TaKaRa, Kusatsu shiga, Japan) and gDNA Eraser Kit according to the manufacturer’s instructions, and the samples were stored in a refrigerator at −20 °C for later use.

### 2.5. Primer Design and Quantitative Real-Time Polymerase Chain Reaction (RT-qPCR)

In the *Heortia vitessoides* Moore transcriptome database, we obtained the full-length cDNA sequences of genes involved in chitin synthesis pathway: Tre (Trehalase), Hk (Hexokinase), GPI (glucose-6-phosphate isomerase), GFAT (glutamine fructose-6-phosphate aminotransferase), GNA (glucosamine 6-phosphate N-acetyltransferase), UAP (UDP-N-acetylglucosamine pyrophosphorylase), CHSA (chitin synthase A), CHSB (chitin synthase B), and CHT (chitinase). Specific primers were designed within the conserved regions using Primer Premier 5.0 software (Premier Biosoft International, Palo Alto, CA, USA), and the synthesis of primers was outsourced to Guangzhou TsingkeBiotechnology Co., Ltd. (TsingkeBiotechnology Co., Ltd., Guangzhou, China) The primer sequences are shown in Table 1.

The previously synthesized cDNA templates were diluted to prepare RT-qPCR reaction templates. Fluorescence quantitative analysis was performed using the LightCycler 480 II Real-Time PCR System. Three technical replicates were established, with β-actin used as the reference gene.

### 2.6. dsRNA Preparation and Injection

The synthesis of dsRNA was conducted using the T7 RiboMAXTM Express RNAi System kit. Primers containing T7 RNA polymerase promoter sequences were synthesized, and PCR was performed to obtain DNA templates. Subsequently, dsHvCP3L and dsGFP segments were synthesized. After removing the DNA template, the dsRNA annealing and single-stranded RNA (ssRNA) were eliminated, followed by purification of the dsRNA. The purified dsRNA was diluted with nuclease-free water and quantified using an Implen NanoPhotometer. The dsRNA was diluted to a concentration of 5 µg/µL, and 1 µL was injected into the dorsal side of the penultimate abdominal segment of each larva using a microinjection needle. For the control group, dsGFP and DEPC were used at the same concentration and dosage. Each group consisted of at least 30 larvae, and phenotypic changes and survival rates were recorded for 4 groups during the observation period. Phenotypic changes were assessed by touching the larvae with a brush, and larvae showing no response within one minute were considered dead.

### 2.7. Methoxyfenozide Stress Experiment and Paraffin Section

To prepare the Methoxyfenozide solution (Dow AgroSciences, Zionsville, IN, USA), we diluted the Methoxyfenozide solution in a container with a 0.1% Triton X-100 aqueous solution to the experimental concentration. Using the leaf-dipping method, we submerged 10 fresh Aquilaria sinensis leaves in the solution for 10 s and then removed them. A 0.1% Triton X-100 aqueous solution was used as the control. After air-drying the treated leaves naturally, they were used to feed 30 L4 stage *Heortia vitessoides* Moore larvae, with each experimental group repeated three times.

At specified time points post-treatment (12, 24, 36, 48, and 72 h), larvae samples were collected. Additionally, larvae of *Heortia vitessoides* Moore raised under normal conditions during the same period were collected as the control group. All collected samples were rapidly frozen and stored in an ultra-low temperature freezer (−80 °C).

### 2.8. Statistical Analysis

The experimental data were preliminarily analyzed using Excel software (MicrosoftExcel 2016 MsO (16.0.8827.2131) 32bit). The relative expression levels of the target gene were calculated using the 2^−ΔΔCt^ method [35]. Subsequently, one-way analysis of variance (ANOVA) was performed using SPSS 18.0 software (IBM, Armonk, NY, USA) to analyze the differences among different developmental stages and tissues, followed by Tukey’s test for post-hoc analysis. Statistical significance was considered when *p* < 0.05. The data are presented as mean ± standard error.

## 3. Results

### 3.1. Sequence Analysis of HvCP3L and Phylogenetic Analysis

**Figure 1:**
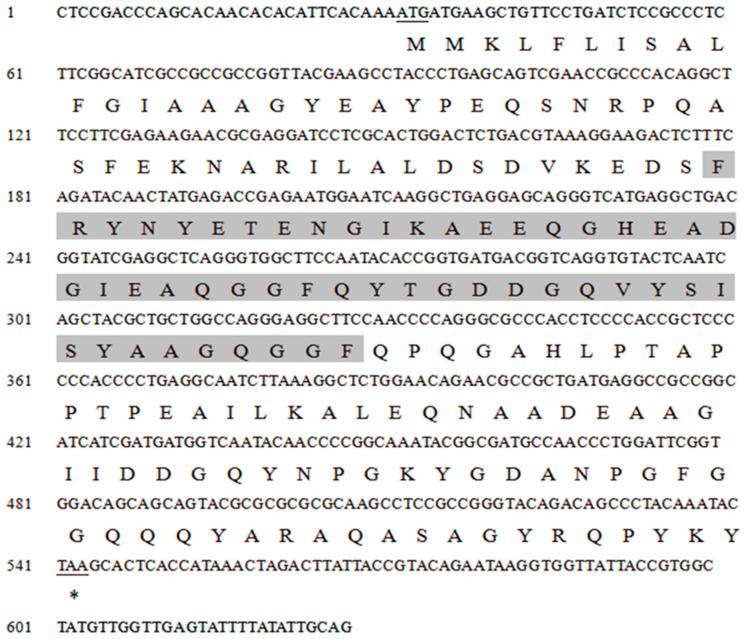
The amino acid sequence of HvCP3L from *Heortia vitessoides* Moore. The start codon and the termination codon are marked with underlined, conserved regions are marked with gray shading, “*” Representing termination codons.

The gene sequence was retrieved through transcriptome analysis. The complete sequence of the CP3L gene, named HvCP3L (GenBank accession number: PP468568), was obtained through homology search using BLAST on the NCBI website. The sequence is 628 bp in length, with an open reading frame (ORF) of 513 bp, encoding 170 amino acids. CD-search on NCBI revealed that the amino acid sequence of HvCP3L contains the conserved domain Chitin Bind 4. Chitin Bind 4 is a chitin-binding domain of the chitin-binding superfamily found in arthropod cuticular proteins, indicating that HvCP3L belongs to the insect cuticular protein CPR family (Figure 1).

**Figure 2:**
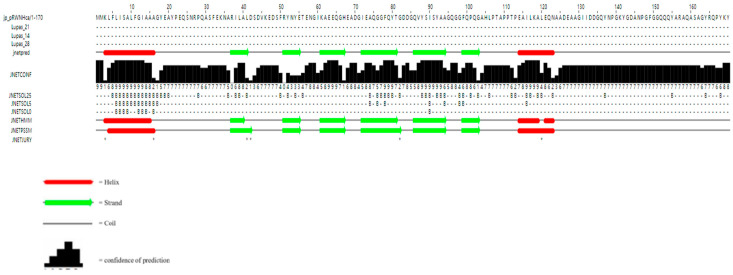
Secondary structure prediction of HvCP3L.

**Figure 3:**
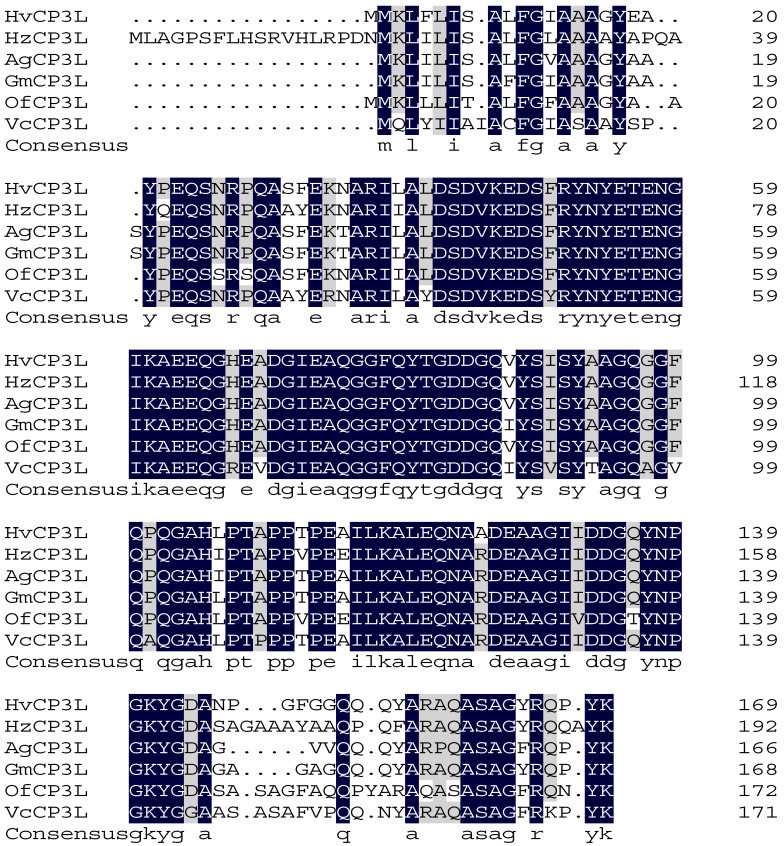
Sequence alignment of HvCP3L with insect homologs. The amino acid residues thatare identical in all sequences are marked with dark shading, whereas light shading indicates thatat least 75% amino acids are identical in all sequences. The aligned sequences are the predictedThe aligned sequences are the predicted amino acid sequences of CP3L from Heortia vitessoides Moore (HvCP3L WWZ69572.1), Helicoverpa zea (HzCP3L XP_047031077.1), Achroia grisella (XP_059057921.1), Galleria mellonella (XP_026763387.1), Ostrinia furnacalis (OfCP3LXP_028179309.1), Vanessa cardui (VcCP3L XP_046970535.1).

**Figure 4:**
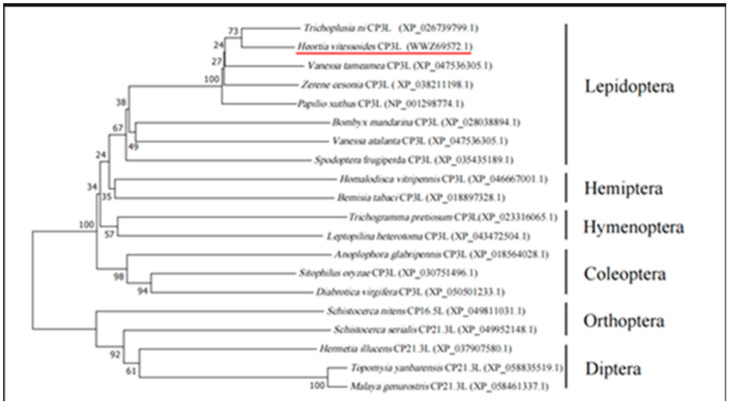
Phylogenetic analysis of HvCP3L. The predicted amino acid sequences of HvCP3L together with 19 selected CP members were aligned, and a phylogenetic tree was constructed using MEGA7. The CP3L of Heortia vitessoides Moore marked with red underline. GenBank accession numbers are as follows:Trichoplusia ni CP3L (XP_026739799.1); Heortia vitessoides CP3L (WWZ69572.1); Vanessa tameamea CP3L (XP_047536305.1); Zerene cesonia CP3L (XP_038211198.1); Papilio xuthus CP3L (NP_001298774.1); Bombyx mandarina CP3L (XP_028038894.1); Vanessa atalanta CP3L (XP_047536305.1); Spodoptera frugiperda CP3L (XP_035435189.1); Homalodisca vitripennis CP3L (XP_046667001.1); Bemisia tabaci CP3L (XP_018897328.1); Trichogramma pretiosum CP3L(XP_023316065.1); Leptopilina heterotoma CP3L (XP_043472504.1); Anoplophora glabripennis CP3L (XP_018564028.1); Sitophilus oryzae CP3L (XP_030751496.1); Diabrotica virgifera CP3L (XP_050501233.1); Schistocerca nitens CP16.5L (XP_049811031.1); Schistocerca serialis CP21.3L (XP_049952148.1); Hermetia illucens CP21.3L (XP_037907580.1); Topomyia yanbarensis CP21.3L (XP_058835519.1); Malaya genurostris CP21.3L (XP_058461337.1).

Using the ExPASyProtParam (http://web.expasy.org/protparam (accessed on 12 June 2023)) tool, the theoretical molecular weight of the protein encoded by the gene was predicted to be 18.30 kDa, with a predicted isoelectric point of 4.48. It contains 23 negatively charged amino acid residues (Asp and Glu) and 12 positively charged amino acid residues (Arg and Lys). Analysis using NetNGlyc 1.0 Server showed that HvCP3L lacks transmembrane domains and potential N-glycosylation sites. Prediction using the Jpred online tool revealed that the amino acid sequence of HvCP3L contains two helical structures, with the remaining parts alternating between β-sheets and random coils. Previous studies have suggested that the conserved regions of insect cuticular proteins may be rich in β-sheets (Figure 2). Consistent with this, our prediction for the secondary structure of HvCP3L revealed frequent occurrence of β-sheets in the conserved region. Furthermore, we analyzed the amino acid sequence of HvCP3L using the insect cuticular protein database (http://aias.biol.uoa.gr/CutProtFam-Pred/search.php (accessed on 3 August 2023)), which indicated that HvCP3L belongs to the RR-1 type cuticular protein. We downloaded amino acid sequences of CP3L from five insects (*Helicoverpa zea*, *Achroia grisella*, *Galleria mellonella*, *Ostrinia furnacalis*, and *Vanessa cardui*) from GenBank. The similarity values of these insect CP3L sequences to HvCP3L were found to be 77.20%, 90.64%, 92.98%, 86.71%, and 78.61%, respectively (Figure 3). To understand the relationship between CP3L genes and different insects, we constructed a phylogenetic tree using CP3L genes from Lepidoptera, Coleoptera, Hymenoptera, Hemiptera, Orthoptera, and Diptera. The results showed that HvCP3L clustered with other Lepidoptera insects, such as *Trichoplusia ni* and *Vanessa tameamea* (Figure 4).

### 3.2. Stage-Specific and Tissue-Specific Expression Patterns of HvCP3L

**Figure 5:**
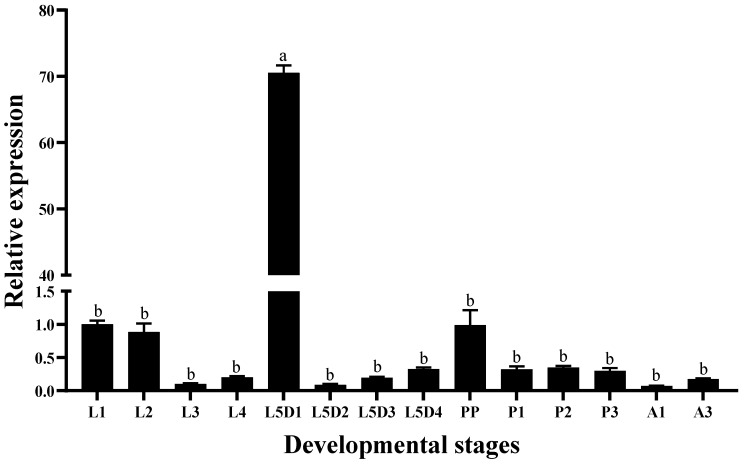
Relative expression levels of HvCP3L at different stages: L1–L4, first-to fourth-instar larvae; L5D1-L5D4, 1-to-4-day fifth-instar larvae; PP, pre-pupae; P1–P3, 1-to-3-day-old pupae; A1, 1-day-old adults; A3, 3-day-old adults. Error bars represent mean ± standard error of three biological replicates. Different letters above error bars indicate significant differences (*p* < 0.05) based on one-way ANOVA and Tukey’s test.

**Figure 6:**
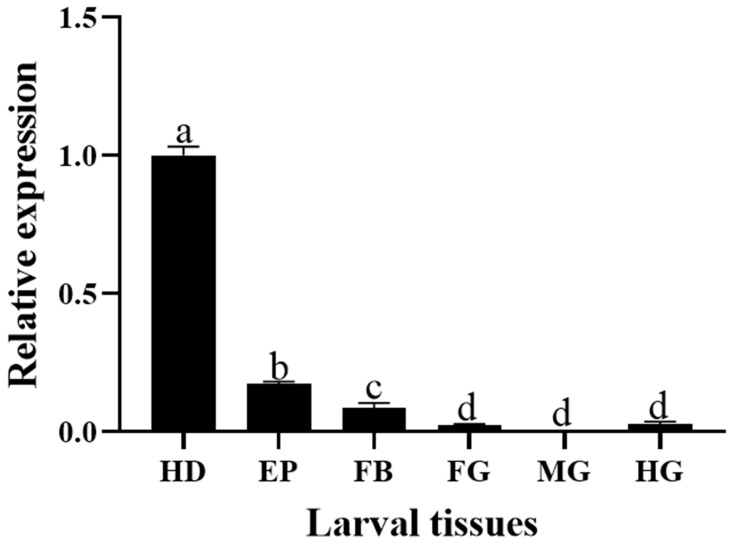
Relative expression levels of HvCP3L in different larval tissues (tissue anatomy for the fifth-instar larvae). Relative expression in larval tissues: HD, head; EP, epidermis; FG, foregut; MG, midgut; HG, hindgut; and FB, fat body. Error bars represent mean ± standard error of three biological replicates Different letters above error bars indicate significant differences (*p* < 0.05), which were based on one-way analysis of variance (ANOVA) and Tukey’s test.

**Figure 7:**
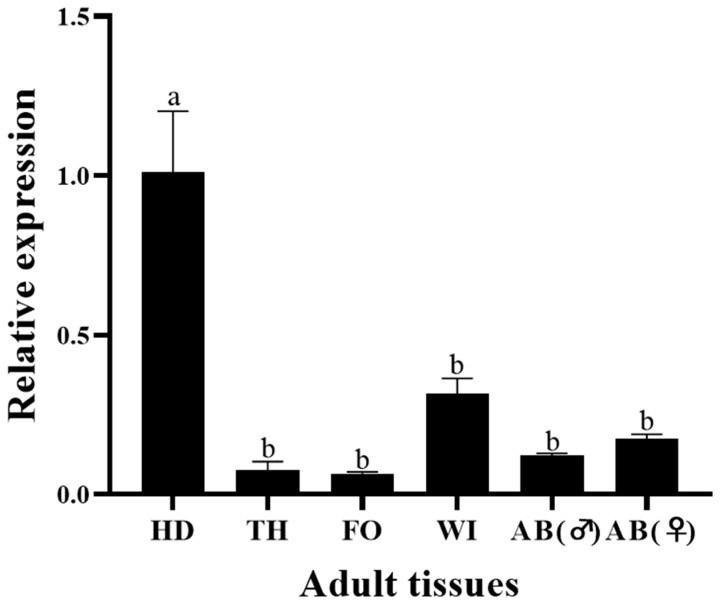
Relative expression in adult tissues: HD, head; TH, thorax; AB(♂), male abdomen; AB(♀), female abdomen; FO, foot; and WI, wing. Error bars represent mean ± standard error of three biological replicates. Different letters above error bars indicate significant differences (*p* < 0.05), which were based on one-way analysis of variance (ANOVA) and Tukey’s test.

We used the RT-qPCR method to investigate the relative expression pattern of HvCP3L in different developmental stages and tissues of *Heortia vitessoides* Moore. Our results revealed that HvCP3L expression was detected from larval to adult stages of *Heortia vitessoides* Moore. Specifically, during the larval stages, HvCP3L showed higher expression levels at L1, L2, and L5D1 stages, with significantly elevated expression at the L5D1 stage, reaching 70 times higher than that at the L1 stage. Moreover, HvCP3L expression was also higher during the pre-pupal stage compared to the pupal stage. In the adult stage, expression at the A3 stage was higher than that at the A1 stage. Analysis of expression levels in six different larval tissues, including the head, epidermis, foregut, midgut, hindgut, and fat body, revealed significant differences in HvCP3L expression among tissues (Figure 5). Particularly, the highest expression was observed in the larval head, while expression in the foregut and hindgut was relatively weak, and almost absent in the midgut (Figure 6). In adult tissues, HvCP3L expression was significantly higher in the head and wings compared to other tissues, with the head exhibiting the highest expression. Additionally, expression levels in the female abdomen were slightly higher than those in the male abdomen, suggesting a potential involvement of HvCP3L in female reproductive development (Figure 7).

### 3.3. Silencing of HvCP3L via RNAi

**Figure 8:**
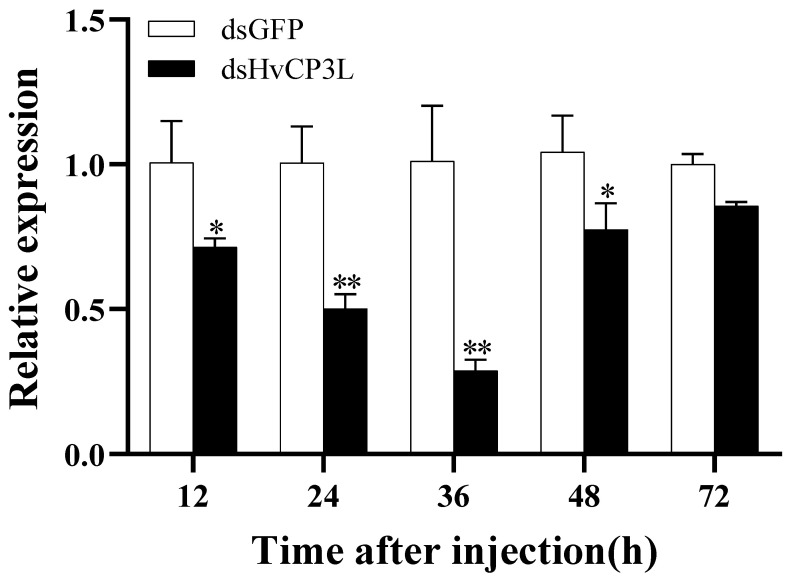
Changes in mRNA level after treatment with specific RNA interference. Relative transcript levels of HvCP3L in L4 larvae after injection with dsHvCP3L at a concentration of 5.0 µg/µL for 12, 24, 36, 48, 72 h. The sample size was 120 larvae, which were divided into three biological replicates. Error bars represent mean ± standard error of three biological replicates. * *p* < 0.05, ** *p* < 0.01. Analysis was performed via one-way analysis of variance (ANOVA), followed by Student’s *t*-test.

We injected dsHvCP3L into L4 larvae and studied the RNAi-mediated silencing of HvCP3L expression levels using RT-qPCR (Figure 8). The results indicated that dsHvCP3L effectively silenced the target gene, with HvCP3L expression levels lower than the control group at 12, 24, 36, 48, and 72 h post dsRNA injection. At 36 h post-injection, HvCP3L expression was downregulated to 27% of the control group, indicating the highest interference efficiency at this time point.

### 3.4. The Effect of RNAi Silencing HvCP3L on Chitin Synthesis Pathway

**Figure 9:**
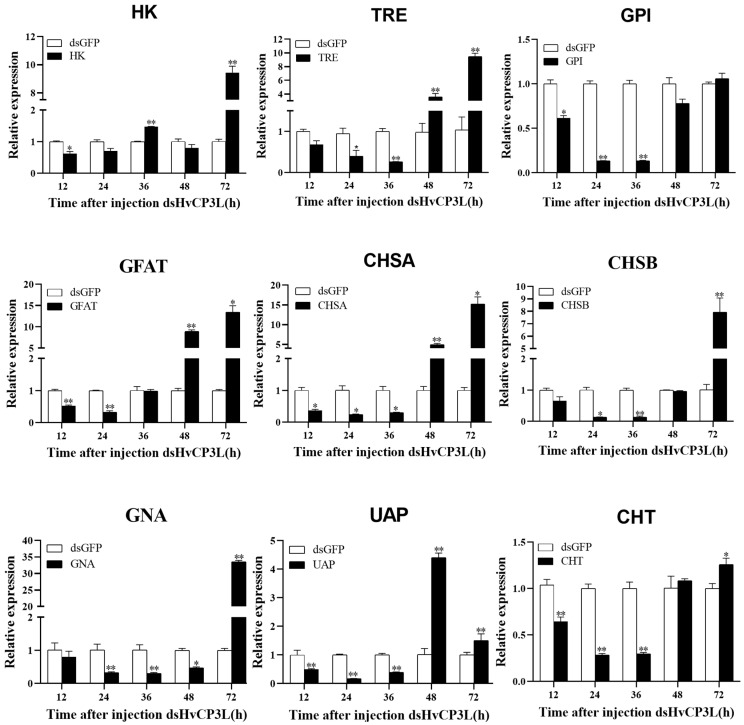
The changes in chitin pathway level after RNA interference of HvCP3L gene. Error bars represent mean ± standard error of three biological replicates. Different letters above error bars indicate significant differences (*p < 0.05, ** *p* < 0.01), which were based on one-way analysis of variance (ANOVA) and Tukey’s test.

We collected samples of larvae after dsHvCP3L injection and used RT-qPCR technology to detect the relative transcription levels of chitin pathway genes. We found that knocking down HvCP3L could affect other genes in the chitin signaling pathway of *Heortia vitessoides* Moore (Figure 9). The expression patterns of most chitin pathway genes showed a similar trend to that of HvCP3L, with expression initially decreasing after knockdown and then gradually increasing. Within the first 36 h post dsHvCP3L injection, except for the HK gene, which showed a sudden increase in expression to 1.4 times that of the control group at 36 h, the expression levels of the other chitin pathway genes were suppressed. The expression levels of TRE and GNA genes were similar to HvCP3L, reaching their lowest point at 36 h, while the expression levels of CHT, GPI, CHSA, CHSB, UAP, and GFAT genes reached their lowest point at 24 h, earlier than the changes in HvCP3L expression. Interestingly, except for the HK gene, which showed a decrease in expression to 0.8 times that of the control group at 48 h, the expression levels of other genes started to increase at 48 h, and even significantly exceeded those of the control group.

### 3.5. The Phenotypic Analysis and Survival Assay after RNAi

**Figure 10:**
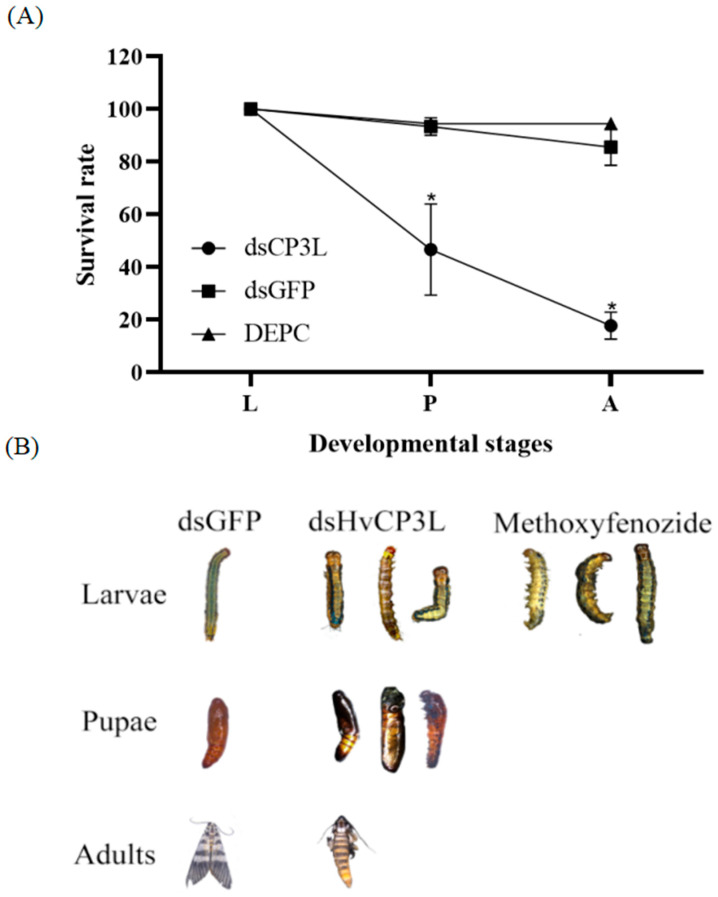
Analysis after interference with HvCP3L and methoxyfenozide treatment. (**A**) Effects of HvCP3L RNAi on larval-to-pupal and pupal-to-adult transition rates. Rates of insect survival from fifth-instar larval stage to adulthood after dsHvCP3L injection (* *p* < 0.05, Kaplan–Meier survival analysis with log-rank test). Data are the mean ± standard error of three biological repeats. (**B**) Phenotypic abnormalities caused by RNAi interference with HvCP3L and methoxyfenozide treatment.

After successfully silencing HvCP3L, we compared it with the dsGFP and DECP injection groups. The results showed that individuals injected with dsHvCP3L exhibited extremely high mortality rates and developmental abnormalities. The survival rate from larvae to adults was only 17.7%, significantly lower than that of the control group (Figure 10).

### 3.6. Effects of Methoxyfenozide on HvCP3L and Phenotype

**Figure 11:**
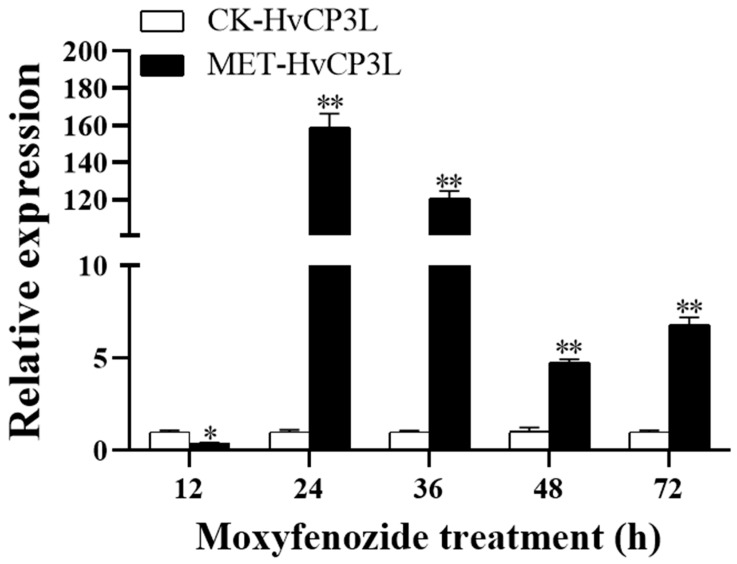
Changes in mRNA level after treatment with methoxyfenozide. The CK indicated that the experimental control was fed without methoxyfenozide. Error bars represent mean ± standard error of three biological replicates. * *p* < 0.05, ** *p* < 0.01. Analysis was performed via one-way analysis of variance (ANOVA), followed by Student’s *t*-test.

In the stress experiment with L4 *Heortia vitessoides* Moore using methoxyfenozide, we observed that, 12 h later, the expression level of the HvCP3L gene was downregulated to 38.7%. However, at 24 h and 36 h, the expression levels of HvCP3L were 158-fold and 120-fold higher than the control group, respectively, significantly higher than the control group, and most larvae died during the entire period. At 48 h and 72 h, the relative expression levels of HvCP3L remained 4.8-fold and 6.8-fold higher than the control group, respectively (Figure 11).

After methoxyfenozide treatment, noticeable darkening and wrinkling of the cuticle were observed in L4 *Heortia vitessoides* Moore larvae.

## 4. Discussion

Insect cuticular proteins play crucial roles in the growth and development of insects. Serving as major components of insect cuticles, they are vital for the formation and development of insect epidermis [36]. The cross-linking interactions between different types of cuticular proteins confer distinct physicochemical properties to the insect cuticle, thereby influencing the process of insect resistance formation [37,38]. In this study, we successfully identified a cuticular protein 3-like gene (HvCP3L) from the existing transcriptome of *Heortia vitessoides* Moore. In amino acid sequence analysis, HvCP3L encodes a protein sequence with high similarity to CP3L in Lepidoptera. It exhibits the highest homology with *Galleria mellonella* GmCP3L, reaching 92.98%. The phylogenetic tree constructed using amino acid sequences demonstrates that HvCP3L is most closely related to other Lepidopteran insects, showing lower homology with CP3L from Coleoptera, Diptera, Hemiptera, and Hymenoptera.

The formation and differentiation of the insect cuticle commence in the egg and are completed during each molting cycle, involving the dissolution of the old cuticle and the construction of a new one, a process that spans the entire insect’s lifecycle. In studies of cuticular protein genes in *Heliothis armigera*, SgLCP17 and SgAbd5 exhibit higher expression levels during the larval stage, while expression is nearly absent in adults and pupae. Additionally, the expression level of the SgLCP17 gene significantly increases in the late fourth instar and reaches its highest level during the pre-pupal stage [39]. In *Anopheles gambiae*, four CPF and one CPFL cuticular protein genes exhibit higher expression levels before pupation and eclosion, while other CPFL2-7 genes are highly expressed during the larval period [15]. We found that the HvCP3L gene is expressed at various larval stages of *Heortia vitessoides* Moore and shows a certain degree of periodicity. Expression is higher in early larvae (L1, L2), significantly lower in L3 compared to the previous two instars, but gradually increases thereafter. Expression is significantly higher at L5D1 than at other larval stages, but decreases significantly at L5D2, followed by an increase until the pre-pupal stage (PP), similar to the expression pattern in L3. This finding is similar to the expression pattern of LmAbd-2 in *Locusta migratoria* cuticular protein research, which shows periodic high expression during cuticle formation at various larval stages [40]. The periodic changes in HvCP3L gene expression mainly occur between the immediate post-molt of L4 and L5D1, possibly due to changes in insect hormone titers involved in the formation and development of the *Heortia vitessoides* Moore cuticle.

The expression of cuticular protein genes also exhibits tissue specificity. In the R&R type cuticular proteins, the CPR family can be divided into three subfamilies based on differences in the type and location of the cornified layer. Members of the RR-1 subfamily are mainly highly expressed in less cornified epidermis, while RR-2 subfamily members are mainly expressed in highly cornified epidermis, and RR-3 subfamily members may be involved in the formation of new cuticle after molting. In *Anopheles gambiae*, CPR family RR-1 and RR-2 subfamily members are localized in the cuticle, and RR-1 subfamily cuticular proteins such as AmCPR12, AmCPR13, AmCPR22, AmCPR61, AmCPR133, and AmCPR153 are found in the soft intersegmental membranes of fourth instar larvae. AmCPR75 is found in the intersegmental membranes of adults. RR-2 subfamily cuticular proteins are found in the original cuticle layer of pupae and the hardened cuticle of adult legs [41]. In *Apis cerana*, the AccCPR2 gene is highly expressed in the cuticle, thorax, wings, and legs, and high expression of AccCPR2 is also observed in honeybee stings [42]. We found that, although the HvCP3L gene belongs to the RR-1 subfamily, its expression in hard tissues is higher than in soft tissues. Expression in the larval head is significantly higher than in the cuticle. Moreover, expression in the head and wings of adults is higher than in other adult tissues, which differs from the results of studies in *Anopheles gambiae* [43]. However, in the study of cuticular proteins in *Cydia pomonella*, RR-1 subfamily genes are also present in small amounts in hard tissues, such as the hardened antennae of adults [44]. We speculate that HvCP3L may be associated with other RR-2 subfamily genes during the molting process, but further research is needed.

Previous studies have demonstrated the significant role of cuticular protein genes in cuticle development, and their knockdown can lead to abnormal insect phenotypes and increased mortality rates. In research on *Blattella germanica*, silencing of BgCPLCP1 resulted in a notable increase in wing defects in adults, potentially allowing insecticides to penetrate the exposed wax layer of the cuticle, leading to higher mortality rates and decreased ecological adaptability [45]. Interference with cuticular protein Cpr21L in *Nilaparvata lugens* nymphs led to a high mortality rate and significantly affected testis development, severely impairing the reproductive capacity of male adults [46]. In this study, we injected 1 µL of dsRNA at a concentration of 5 µg/µL into L4 larvae. The results showed that the relative expression level of RNAi reached its lowest point 36 h after dsHvCP3L injection, after which it began to increase. This indicates that RNAi has the expected effect of inhibiting HvCP3L expression. Upon silencing the HvCP3L gene, we observed shortened body length and yellowing or darkening of the cuticle in treated larvae. Some larvae exhibited molting abnormalities, leading to premature death before pupation, and, during the pupal stage, malformed pupae and inability to molt the head capsule were observed. Similar pupal deformities were found in *Drosophila melanogaster*, where the DmCPAP3-E gene regulates chitin arrangement; lack of CPAP3-E function results in abnormal metamorphosis of the cuticle, with the cuticle becoming branched [47]. Furthermore, microscopic observations revealed that knockdown of the HvCP3L gene resulted in a decrease in the thickness of the larval abdominal cuticle, disorganized arrangement of epidermal cells, and separation between the cuticle and epidermal cells. We speculate that the HvCP3L gene may be involved in the formation of the larval cuticle structure, but further investigation is needed to elucidate the underlying mechanism. Similar phenomena were observed in *Tribolium castaneum*, where knockdown of TcCPR69 led to disordered cell arrangement and increased apoptotic cells in the newly formed adult abdominal cuticle [48].

In a study on *Nilaparvata lugens*, it was found that knockdown of the HK gene led to a significant decrease in the expression of chitin pathway-related genes [49]. In this study, interference with HvCP3L resulted in a significant inhibition of the expression of chitin pathway-related genes, including Tre, Hk, GPI, GFAT, GNA, UAP, CHSA, CHSB, and CHT. When the relative expression level of HvCP3L began to increase at 72 h, the expression levels of chitin metabolism pathway genes significantly increased. We speculate that this might be due to compensatory increases in gene expression levels after chitin synthesis was inhibited. Among these genes, the expression patterns of HK and UAP differed from the others. In *Diaphorina citri*, after oral administration of dsDcHK, the expression level of dsDcHK was lowest at 48 h and sharply increased at 72 h. The expression pattern of HvUAP was similar to that of *Diaphorina citri*. After inhibition of HvHK expression, HvUAP expression sharply increased at 48 h [50].

Methoxyfenozide acts as an antagonist to insect ecdysteroids, competing with endogenous 20-hydroxyecdysone (20E) for binding to ecdysone receptors, thereby disrupting the normal expression of downstream genes in the 20E pathway and hindering insect molting. Studies on *Heliothis armigera* have demonstrated that methoxyfenozide inhibits molting and reduces ecdysteroid titers [51]. In our study, after treating L4 *Heortia vitessoides* Moore larvae with methoxyfenozide, we observed significant darkening and wrinkling of the larval cuticle. Additionally, microscopic observations revealed detachment between the larval epidermis and the cuticle, disrupting the formation of the new cuticle during molting. Similar effects have been observed in *Spodoptera litura*, where methoxyfenozide treatment significantly prolongs larval and pupal development, inhibits chitin synthesis, leads to the failure of new cuticle formation, disrupts molting, and ultimately results in larval mortality [52,53].

Furthermore, we noticed a significant upregulation of HvCP3L expression levels 24 h after methoxyfenozide treatment, surpassing those in the control group. This phenomenon closely resembles findings in *Apis mellifera* and *Apis cerana*, where high concentrations of molting hormones stimulate the expression of cuticular protein genes. Conversely, during the late stages of molting when ecdysteroid concentrations decline, the expression of cuticular protein genes such as AmelCPR14 and AccCPR1 begins to increase [54,55].

## Figures and Tables

**Table 1:** Primers used for RT-qPCR and synthesis of dsHvCP3L and dsGFP.

Primer Name	Forward (5′-3′)	Reverse (5′-3′)	TM	Product Length (bp)
TRE	CGCCACTGCTGACTGCTATG	GCCTTCGGTCGTCGGTATC	60.0/60.1	249
HK	CCAGACCTGCTTATCAATACC	GCCGAATAGAAGACCCATC	54.2/54.3	207
GPI	CGGGCAGTGGAAAGGGTA	TCAGGACTTCGGCTAAATGG	58.9/57.8	173
GFAT	CGAGTTGTCGGTTGAAGAATG	CGTTGCGGATGCGAGTTA	58.0/58.2	102
GNA	TTGGAGGATGTCGTGGTTA	ATTCATAGCGTTAGAGTTGCC	54.8/54.4	191
UAP	TTCCGAAGTCAACGAAACA	AGAAATCTCCTCCAAACCAAT	54.4/54.8	169
CHSA	ATTGCCTTTGTATAATACCTGC	CCTATCGGACTCTGTCTTGTT	54.1/53.5	232
CHSB	AAGCAACAGCATTCGTCGTG	CCGTAGCAATCCGAGTGAAA	59.5/58.7	300
CHT	AAGGACGGAAAGACGGGATT	TATGGGATGGCGGAGTAGATG	60.1/60.0	170
HvCP3L	CTCCGACCCAGCACAACAC	CCTCAGCCTTGATTCCATTC	59.0/58.0	220
T7 + dsGFP	taatacgactcactatagggCAGTTCTTGT TGAATTAGATG	taatacgactcactatagggTTTGGTTTGT CTCCCATGATG	71.5/75.5	400
T7 + dsHvCP3L	taatacgactcactatagggATACAACTATGAGACCGAGAATG	taatacgactcactatagggGCTGCTGTCCACCGAAT	74.4/77.9	308
β-actin	GTGTTCCCCTCTATCGTGG	TGTCGTCCCAGTTGGTGAT	57.32/55.11	119

## Data Availability

The data presented in this study are available upon request from the corresponding author.

## References

[1] Locke M. (2001). The Wigglesworth Lecture: Insects for studying fundamental problems in biology. J. Insect Physiol..

[2] Oluwatobi B., Oksana S., Aleksei G., Jouni S. (2020). Benefits of insect colours: A review from social insect studies. Oecologia.

[3] Moussian B. (2010). Recent advances in understanding mechanisms of insect cuticle differentiation. Insect Biochem. Mol. Biol..

[4] Snyder M.P., Kimbrell D., Hunkapiller M., Hill R., Fristrom J., Davidson N. (1982). A transposable element that splits the promoter region inactivates a *Drosophila* cuticle protein gene. Proc. Natl. Acad. Sci. USA.

[5] Futahashi R., Okamoto S., Kawasaki H., Zhong Y.-S., Iwanaga M., Mita K., Fujiwara H. (2008). Genome-wide identification of cuticular protein genes in the silkworm, *Bombyx mori*. Insect Biochem. Mol. Biol..

[6] Ioannidou Z.S., Theodoropoulou M.C., Papandreou N.C., Willis J.H., Hamodrakas S.J. (2014). CutProtFam-Pred: Detection and classification of putative structural cuticular proteins from sequence alone, based on profile Hidden Markov Models. Insect Biochem. Mol. Biol..

[7] Olga V., Zhe L., Jie D., Hong J., Zhen Z. (2019). Identification and temporal expression profiles of cuticular proteins in the endoparasitoid wasp, *Microplitis mediator*. Insect Sci..

[8] Rebers J.E., Riddiford L.M. (1988). Structure and expression of a Manduca sexta larval cuticle gene homologous to *Drosophila* cuticle genes. J. Mol. Biol..

[9] Andersen S.O. (2000). Studies on proteins in post-ecdysial nymphal cuticle of locust, *Locusta migratoria*, and cockroach, *Blaberus craniifer*. Insect Biochem. Mol. Biol..

[10] Jasrapuria S., Specht C.A., Kramer K.J., Beeman R.W., Muthukrishnan S. (2012). Gene Families of Cuticular Proteins Analogous to Peritrophins (CPAPs) in *Tribolium castaneum* Have Diverse Functions. PLoS ONE.

[11] Cornman R.S., Willis J.H. (2009). Annotation and analysis of low-complexity protein families of *Anopheles gambiae* that are associated with cuticle. Insect Mol. Biol..

[12] Zhou D., Duan B., Sun Y., Ma L., Zhu C., Shen B. (2017). Preliminary characterization of putative structural cuticular proteins in the malaria vector *Anopheles sinensis*. Pest Manag. Sci..

[13] Chen E.-H., Hou Q.-L., Dou W., Wei D.-D., Yue Y., Yang R.-L., Yang P.-J., Yu S.-F., De Schutter K., Smagghe G. (2018). Genome-wide annotation of cuticular proteins in the oriental fruit fly *Bactrocera dorsalis*, changes during pupariation and expression analysis of CPAP3 protein genes in response to environmental stresses. Insect Biochem. Mol. Biol..

[14] Vannini L., Bowen J.H., Reed T.W., Willis J.H. (2015). The CPCFC cuticular protein family: Anatomical and cuticular locations in *Anopheles gambiae* and distribution throughout Pancrustacea. Insect Biochem. Mol. Biol..

[15] Togawa T., Dunn W.A., Emmons A.C., Willis J.H. (2007). CPF and CPFL, two related gene families encoding cuticular proteins of *Anopheles gambiae* and other insects. Insect Biochem. Mol. Biol..

[16] Guan X., Middlebrooks B.W., Alexander S., Wasserman S.A. (2006). Mutation of TweedleD, a member of an unconventional cuticle protein family, alters body shape in *Drosophila*. Proc. Natl. Acad. Sci. USA.

[17] Dittmer N.T., Tetreau G., Cao X., Jiang H., Wang P., Kanost M.R. (2015). Annotation and expression analysis of cuticular proteins from the tobacco hornworm, *Manduca sexta*. Insect Biochem. Mol. Biol..

[18] Dong Z., Zhang W., Zhang Y., Zhang X., Zhao P., Xia Q. (2016). Identification and Characterization of Novel Chitin-Binding Proteins from the Larval Cuticle of Silkworm, *Bombyx mori*. J. Proteome Res..

[19] Liang Q., Gao X., Ri-xin W., Song-zhen H., Jie C., Xiao-ling T., Hai H., Chun-lin L., Ting-ting G., Ya-qun X. (2014). Mutation of a cuticular protein, BmorCPR2, alters larval body shape and adaptability in silkworm, *Bombyx mori*. Genetics.

[20] Arakane Y., Lomakin J., Gehrke S.H., Hiromasa Y., Tomich J.M., Muthukrishnan S., Beeman R.W., Kramer K.J., Kanost M.R. (2012). Formation of Rigid, Non-Flight Forewings (Elytra) of a Beetle Requires Two Major Cuticular Proteins. PLoS Genet..

[21] Hou Q.-L., Chen E.-H., Dou W., Wang J.-J. (2021). Knockdown of specific cuticular proteins analogous to peritrophin 3 genes disrupt larval and ovarian development in *Bactrocera dorsalis* (*Diptera: Tephritidae*). Insect Sci..

[22] Zhao X., Su Y., Shao T., Fan Z., Cao L., Liu W., Zhang J. (2022). Cuticle protein gene LmCP8 is involved in the structural development of the ovipositor in the migratory locust *Locusta migratoria*. Insect Mol. Biol..

[23] Okamoto S., Futahashi R., Kojima T., Mita K., Fujiwara H. (2008). Catalogue of epidermal genes: Genes expressed in the epidermis during larval molt of the silkworm *Bombyx mori*. BMC Genom..

[24] Sapin G.D., Tomoda K., Tanaka S., Shinoda T., Miura K., Minakuchi C. (2020). Involvement of the transcription factor E75 in adult cuticular formation in the red flour beetle *Tribolium castaneum*. Insect Biochem. Mol. Biol..

[25] Bouhin H., Braquart C., Charles J.P., Quennedey B., Delachambre J. (1993). Nucleotide sequence of an adult-specific cuticular protein gene from the beetle *Tenebrio molitor*: Effects of 20-hydroxyecdysone on mRNA accumulation. Insect Mol. Biol..

[26] Lemoine A., Mathelin J., Braquart-Varnier C., Everaerts C., Delachambre J. (2004). A functional analysis of ACP-20, an adult-specific cuticular protein gene from the beetle *Tenebrio*: Role of an intronic sequence in transcriptional activation during the late metamorphic period. Insect Mol. Biol..

[27] Dhadialla T.S., Carlson G.R., Le D.P. (1998). New insecticides with ecdysteroidal and juvenile hormone activity. Annu. Rev. Entomol..

[28] Smagghe G., Pineda S., Carton B., Del Estal P., Budia F., Viñuela E. (2003). Toxicity and kinetics of methoxyfenozide in greenhouse-selected *Spodoptera exigua* (*Lepidoptera: Noctuidae*). Pest Manag. Sci..

[29] Schneider M.I., Smagghe G., Pineda S., Viñuela E. (2004). Action of insect growth regulator insecticides and spinosad on life history parameters and absorption in third-instar larvae of the endoparasitoid *Hyposoter didymator*. Biol. Control.

[30] Pineda S., Schneider M.-I., Smagghe G., Martinez A.-M., Del Estal P., Vinuela E., Valle J., Budia F. (2007). Lethal and sublethal effects of methoxyfenozide and spinosad on *Spodoptera littoralis* (*Lepidoptera: Noctuidae*). J. Econ. Entomol..

[31] Schneider M., Smagghe G., Pineda S., Vinuela E. (2008). The ecological impact of four IGR insecticides in adults of *Hyposoter didymator* (Hym., Ichneumonidae):: Pharmacokinetics approach. Ecotoxicology.

[32] Zhou M., Wang H., Suolangjiba, Kou J., Yu B. (2008). Antinociceptive and anti-inflammatory activities of *Aquilaria sinensis* (Lour.) Gilg. Leaves extract. J. Ethnopharmacol..

[33] Wu Y., Li E., Li Y., Wu Q., Tian W., Liu K., Niu Y., Wang D., Liu J.-G., Hu Y. (2016). Iriflophenone Glycosides from *Aquilaria sinensis*. Chem. Nat. Compd..

[34] Qiao H.-L., Lu P.-F., Chen J., Ma W.-S., Qin R.-M., Li X.-M. (2012). Antennal and behavioural responses of *Heortia vitessoides* females to host plant volatiles of *Aquilaria sinensis*. Entomol. Exp. Et Appl..

[35] Livak K.J., Schmittgen T.D. (2001). Analysis of relative gene expression data using real-time quantitative PCR and the 2(-Delta Delta C(T)) Method. Methods.

[36] Moussian B., Seifarth C., Mueller U., Berger J., Schwarz H. (2006). Cuticle differentiation during *Drosophila* embryogenesis. Arthropod Struct. Dev..

[37] Cui L., Yuan H., Yang D., Rui C., Mu W. (2017). The Mechanism by Which Dodecyl Dimethyl Benzyl Ammonium Chloride Increased the Toxicity of Chlorpyrifos to *Spodoptera exigua*. Front. Pharmacol..

[38] Balabanidou V., Grigoraki L., Vontas J. (2018). Insect cuticle: A critical determinant of insecticide resistance. Curr. Opin. Insect Sci..

[39] Zheng J., Wu P., Huang Y., Zhang Y., Qiu L. (2024). Identification of insect cuticular protein genes LCP17 and SgAbd5 from *Helicoverpa armigera* and evaluation their roles in fenvalerate resistance. Pestic. Biochem. Physiol..

[40] Jia P., Zhang J., Yang Y., Liu W., Zhang J., Zhao X. (2019). Expression and function analysis of endocuticle structural glycoprotein gene LmAbd-2 in *Locusta migratoria*. Sci. Agric. Sin..

[41] Vannini L., Willis J.H. (2017). Localization of RR-1 and RR-2 cuticular proteins within the cuticle of *Anopheles gambiae*. Arthropod Struct. Dev..

[42] Tan S., Li G., Guo H., Li H., Tian M., Liu Q., Wang Y., Xu B., Guo X. (2022). Identification of the cuticle protein AccCPR2 gene in *Apis cerana cerana* and its response to environmental stress. Insect Mol. Biol..

[43] Karouzou M.V., Spyropoulos Y., Iconomidou V.A., Cornman R.S., Hamodrakas S.J., Willis J.H. (2007). *Drosophila* cuticular proteins with the R&R Consensus:: Annotation and classification with a new tool for discriminating RR-1 and RR-2 sequences. Insect Biochem. Mol. Biol..

[44] Li Z., Ouyang L., Wu Q., Peng Q., Zhang B., Qian W., Liu B., Wan F. (2024). Cuticular proteins in codling moth (*Cydia pomonella*) respond to insecticide and temperature stress. Ecotoxicol. Environ. Saf..

[45] Cai T., Wang X., Liu B., Zhao H., Liu C., Zhang X., Zhang Y., Gao H., Schal C., Zhang F. (2024). A cuticular protein, BgCPLCP1, contributes to insecticide resistance by thickening the cockroach endocuticle. Int. J. Biol. Macromol..

[46] Chen T., Jiao Q., Ye C., Wu J., Zheng Y., Sun C., Hao P., Yu X. (2023). A Novel Cuticular Protein-like Cpr21L Is Essential for Nymph Survival and Male Fecundity in the Brown Planthopper. Int. J. Mol. Sci..

[47] Tajiri R., Ogawa N., Fujiwara H., Kojima T. (2017). Mechanical Control of Whole Body Shape by a Single Cuticular Protein Obstructor-E in *Drosophila melanogaster*. PLoS Genet..

[48] Xie J., Peng G., Wang M., Zhong Q., Song X., Bi J., Tang J., Feng F., Gao H., Li B. (2022). RR-1 cuticular protein TcCPR69 is required for growth and metamorphosis in *Tribolium castaneum*. Insect Sci..

[49] Pan B.-Y., Li G.-Y., Wu Y., Zhou Z.-S., Zhou M., Li C. (2019). Glucose Utilization in the Regulation of Chitin Synthesis in Brown Planthopper. J. Insect Sci..

[50] Yang S., Zou Z., Xin T., Cai S., Wang X., Zhang H., Zhong L., Xia B. (2022). Knockdown of hexokinase in *Diaphorina citri* Kuwayama (*Hemiptera: Liviidae*) by RNAi inhibits chitin synthesis and leads to abnormal phenotypes. Pest Manag. Sci..

[51] Zhang W., Ma L., Liu X., Peng Y., Liang G., Xiao H. (2021). Dissecting the roles of FTZ-F1 in larval molting and pupation, and the sublethal effects of methoxyfenozide on *Helicoverpa armigera*. Pest Manag. Sci..

[52] Rehan A., Freed S. (2015). Fitness Cost of Methoxyfenozide and the Effects of Its Sublethal Doses on Development, Reproduction, and Survival of *Spodoptera litura* (Fabricius) (*Lepidoptera: Noctuidae*). Neotrop. Entomol..

[53] Zhang G., Zou H., Geng N., Ding N., Wang Y., Zhang J., Zou C. (2020). Fenoxycarb and methoxyfenozide (RH-2485) affected development and chitin synthesis through disturbing glycometabolism in *Lymantria dispar* larvae. Pestic. Biochem. Physiol..

[54] Soares M.P.M., Elias-Neto M., Simoes Z.L.P., Bitondi M.M.G. (2007). A cuticle protein gene in the honeybee: Expression during development and in relation to the ecdysteroid titer. Insect Biochem. Mol. Biol..

[55] Sun R., Zhang Y., Xu B. (2013). Characterization of the response to ecdysteroid of a novel cuticle protein R&R gene in the honey bee, *Apis cerana cerana*. Comp. Biochem. Physiol. B Biochem. Mol. Biol..

